# Rare Variant in the SLC6A2 Encoding a Norepinephrine Transporter Is Associated with Elite Athletic Performance in the Polish Population

**DOI:** 10.3390/genes12060919

**Published:** 2021-06-15

**Authors:** Jakub P. Fichna, Kinga Humińska-Lisowska, Krzysztof Safranow, Jakub G. Adamczyk, Paweł Cięszczyk, Cezary Żekanowski, Mariusz Berdyński

**Affiliations:** 1 Department of Neurodegenerative Disorders, Mossakowski Medical Research Institute, Polish Academy of Sciences, 02-106 Warsaw, Poland; jfichna@imdik.pan.pl (J.P.F.); c.zekanowski@imdik.pan.pl (C.Ż.); 2 Faculty of Physical Education, Gdansk University of Physical Education and Sport, 80-336 Gdansk, Poland; kinga.huminska-lisowska@awf.gda.pl (K.H.-L.); pawel.cieszczyk@awf.gda.pl (P.C.); 3 Department of Biochemistry and Medical Chemistry, Pomeranian Medical University, 70-204 Szczecin, Poland; chrissaf@mp.pl; 4 Department of Theory of Sport, Józef Piłsudski University of Physical Education, 00-968 Warsaw, Poland; jakub.adamczyk@awf.edu.pl

**Keywords:** athletic performance, single nucleotide polymorphism, endurance sports, power sports, combat sports

## Abstract

Numerous genetic factors have been shown to influence athletic performance, but the list is far from comprehensive. In this study, we analyzed genetic variants in two genes related to mental abilities, *SLC6A2 (*rs1805065) and *SYNE1* (rs2635438) in a group of 890 athletes (320 endurance, 265 power, and 305 combat athletes) vs. 1009 sedentary controls. Genotyping of selected SNPs was performed using TaqMan SNP genotyping assays. *SLC6A2* codes for norepinephrine transporter, a protein involved in modulating mood, arousal, memory, learning, and pain perception, while *SYNE1* encodes protein important for the maintenance of the cerebellum—the part of the brain that coordinates complex body movements. Both SNPs (rs2635438 and rs1805065) showed no statistically significant differences between the frequencies of variants in the athletes and the sedentary controls (athletes vs. control group) or in the athlete subgroups (martial vs. control, endurance vs. control, and power vs. control). The rs1805065 T variant of *SLC6A2* was found to be overrepresented in male high-elite martial sports athletes when compared to sedentary controls (OR = 6.56, 95%CI = 1.82–23.59, *p* = 0.010). This supports the hypothesis that genetic variants potentially affecting brain functioning can influence elite athletic performance and indicate the need for further genetic association studies, as well as functional analyses.

## 1. Introduction

The elite athlete status is a complex phenomenon conditioned by numerous interdependent factors, including genetic background. Diverse anthropometrical, physiological and psychological traits contribute to athletic performance and heritability studies show that they are substantially inherited. Between 20 and 90% of the variance in sport-related traits has been proposed to be due to heritable factors. Indeed, until now ca. 200 genomic variants were proposed to be associated with sport performance phenotypes. However, there is insufficient evidence to unequivocally connect specific gene variants with major effects on sport-related phenotypes. This is most probably due to the polygenic nature of complex traits found in sport-related phenotypes. Moreover, the difference between success and failure in sport is often determined by a fraction of a second or a few centimeters. Therefore, even the tiniest genetic contribution to physical or mental abilities can significantly affect the sports level of a particular athlete.

The genetic basis of psychological characteristics predisposing to elite athletic performance has so far been largely neglected and is even less well understood than that of physical abilities. Recently, we proposed that psychological features, such as persistence, patience, mental strength, ambition and pursuance to leadership, and planning skills including stress-coping, preventing anxiety-like behavior, avoiding impulsivity and uncontrolled aggressiveness, can be related to success in sports competition [1].

We selected two genes for association analyses in a large group of athletes and controls. Both genes encode proteins involved in brain functioning and modulation of mental activities that may be important in achieving outstanding athletic results.

*SLC6A2* codes for a norepinephrine transporter (NET) which is involved in modulating mood, arousal, memory, learning, and pain perception. Recently, missense variant (rs1805065, NM_001172504.1:c.296C>T, T99I) in *SLC6A2* was proposed to be associated with athlete status in the Brazilian population [2]. The norepinephrine transporter, also known as solute carrier family 6 member 2 (SLC6A2), is a member of the sodium neurotransmitter symporter family and is responsible for the reuptake of extracellular norepinephrine (NE) into presynaptic nerve terminals and thus is a regulator of NE homeostasis.

Norepinephrine is well known to play an important role in various psychological features such as temperament, impulsiveness, learning, and tolerance to pain. A dysregulation of the NE uptake by NET is associated with diverse neuropsychiatric diseases. For instance, several studies have shown that the *SLC6A2* rs3785143 variant is associated with Attention-Deficit/Hyperactivity Disorder (ADHD) [3,4]. Genetic variations affecting the norepinephrine reuptake can also have a substantial effect on autonomic responses and as a result influence the regulation of blood pressure during exercise [2,5,6]. The existence of variants modulating the influence of norepinephrine on psychological traits is also in line with our previous observation of genetically determined psychological predisposition to athletic achievements [1,7].

The second gene, *SYNE1* codes a protein important for the maintenance of the cerebellum, the part of the brain that coordinates movements. SYNE1 (synaptic nuclear envelope protein 1) also known as enaptin or nesprin-1 is a very large spectrin-repeat-containing protein ubiquitously expressed in a variety of tissues, but critical in the brain where it is especially important for Purkinje cells in the cerebellum. Purkinje cells are GABAergic and inhibitory, and their role is to integrate the activity of both main afferent systems that are also controlled by the numerous inhibitory molecular layer interneurons. One can therefore posit that SYNE1 activity could affect sports performance.

In addition, numerous recessive genetic psychiatric disorders have been associated with SYNE1 deficiency, comprising a phenotypic spectrum ranging from cerebellar ataxia to arthrogryposis multiplex congenita (AMC) [8]. Linkage analysis has identified an association of chromosome region 6q25 (one of the genes in this locus is *SYNE1*) with susceptibility to schizophrenia, depression, and autism [9]. Recently, *SYNE1* intron variant (rs2635438, NM_182961.4:c.23301+3917A>G) was provisionally associated with endurance athlete status at the GWAS level of significance (1.91 × 10^−8^) and in a replication study (OR = 0.132; SE = 0.9004; *p* = 0.024) in the European population [10,11].

In this study, we sought to determine whether the reported associations of *SYNE1* (rs2635438) and *SLC6A2* (rs1805065) variants will be confirmed in a large group of athletes of various sports belonging to the genetically homogeneous Polish population [12]. Both genes and variants can expand the list of genes associated with psychological traits in a genetic profile of top athletic performance proposed by us previously.

## 2. Materials and Methods

The study group comprised 890 elite athletes (646 males—72.6%). The athletes were recruited from various sports and the main inclusion criterion was an outstanding performance at an international or national level, as described previously [1]. The control group comprised 1009 healthy individuals (545 males—54.0%). The characteristics of study groups are presented in Table 1. The geographical distribution of the study participants did not differ systematically between the two groups.

The study participants, both athletes and non-athlete controls, were enrolled independently in two research centers with the same inclusion criteria. All participants were unrelated and of Polish origin. Written consent was obtained from all the participants according to the Declaration of Helsinki (BMJ 1991, 302, 1194). The study was approved by the Ethics Committee of the Józef Piłsudski University of Physical Education in Warsaw in compliance with national legislation and the Code of Ethical Principles for Medical Research Involving Human Subjects of the World Medical Association.

DNA samples were collected between 2010 and 2016. Genomic DNA was extracted from peripheral blood leukocytes using a standard salting-out procedure [13] or from buccal cells collected with the Oragene OG−500 DNA collection kit and using Prep IT L2P purification kit (DNA Genotek Inc., Ottawa, ON, Canada) or High Pure PCR Template Preparation Kit (Roche, Switzerland) according to the manufacturer’s instructions. The latter method was used with athletes unwilling to donate blood samples. DNA samples obtained with both methods were of similar quality. Genotyping of selected SNPs was performed using TaqMan SNP genotyping assays (Assay ID: SLC6A2 rs1805065 C__26354913_10, SYNE1 rs2635438 C__16264777_10) (Life Technologies, Carlsbad, CA, USA) on a StepOne Plus Real-Time PCR system (Life Technologies) and CFX Connect Real-Time Detection System (Bio-Rad, Hercules, CA, USA).

Chi-square test was used for both allelic and genotypic association studies when the number of alleles or genotypes was at least 5 in each group, while Fisher’s exact test was used for tables with lower numbers. The study sample size was sufficient to detect with 80% probability the true effect size for differences in allele frequencies of the two polymorphisms between the athletes and the control group corresponding to OR = 0.20 or 2.32 for rs1805065 and OR = 0.59 or 1.52 for rs2635438. The significance level was set at *p* < 0.05.

## 3. Results

The genotype frequencies of the SNPs in *SYNE1* (rs2635438) and *SLC6A2* (rs1805065) were in accordance with the Hardy–Weinberg equilibrium both in the control and athlete groups (*p* > 0.05). The percentage of missing calls for these SNPs did not exceed 1.6‰. The SNPs showed low polymorphism across the cohort with the MAF of 0.008 for rs1805065 and MAF = 0.037 for rs2635438. Table 2 presents allele and genotype frequency distribution. Minor allele homozygotes of rs2635438 were identified in only two controls.

Both SNPs (rs2635438 and rs1805065) showed no statistically significant differences between the frequencies of variants in the athletes and the sedentary controls (athletes vs. control group) or in the athlete subgroups (martial vs. control, endurance vs. control, and power vs. control).

However, we observed that the T allele of rs1805065 was overrepresented in high elite combat sports athletes when compared to sedentary controls (OR = 3.5211, 95%CI = 1.1618–10.6715, *p* = 0.041). Some minor deviations from the expected (based on null hypothesis) distribution on a point-wise level were also observed after sex stratification. In the male subgroups, we observed statistically significant differences in the dominant model for minor allele T of rs1805065 in the following comparisons: high elite male athletes vs. male control (OR = 3.1637, 95%CI = 1.0138–9.8735, *p* = 0.036) and high elite male martial athletes vs. male control (OR = 6.5576, 95%CI = 1.8228–23.5915, *p* = 0.010). We did not observe any statistically significant association with performance in the female subgroups for both SNP.

The multivariate logistic regression analysis compared high elite martial athletes vs. controls with two independent predictors in a dominant model: sex (which results from the division of players according to the gender criterion) and rs1805065. The presence of T rs1805065 allele is a predictor of high elite combat athlete status independent of sex (Table 3).

## 4. Discussion

The genetic basis of athlete performance is well-established with over 200 variants related to sports achievement reported to date [14,15]. Most of them are common variants with a small phenotypic effect and have been identified using case-control hypothesis-driven association studies. However, a combination of genome-wide association studies (GWAS) and whole genome sequencing (WGS) should soon enable the identification of novel and rare variants. Indeed, a preliminary report on the GWAS approach to detecting novel genetic markers of endurance phenotype, by Al-Khelaifi et al. 2019 has already been published [10].

In the present study, we focused on variants putatively associated with psychical traits important for elite athletic performance. We decided to verify, using a substantially larger group of subject recent reports linking two genes broadly involved in brain functioning with sports performance. Both variants are relatively rare: *SYNE1* rs2635438 with global minor allele frequency MAF = 0.069 and *SLC6A2* rs1805065 MAF = 0.009 according to gnomAD (https://gnomad.broadinstitute.org/ (accessed on 14 April 2021) [16]. Both variants are found at different frequencies in diverse populations all over the world, albeit nowhere are they particularly common. Thus the minor allele frequency of *SYNE1* rs2635438 is from 0.134 in the African/African American population, 0.05 in Latino, to 0.033 in non-Finnish European which is in line with our study (MAF = 0.037). For *SLC6A2* rs1805065, is even rarer but also widespread all over the world, with MAF values ranging from 0.016 in the non-Finnish European, 0.005 in Latino and South Asian, to less than 0.003 in the African/African American as well as the Finnish population. In our combined groups (control and athletes) of Polish origin MAF was 0.008.

For the *SLC6A2* rs1805065, we observed a frequency (MAF = 0.008) similar to that in the study of the Brazilian population (0.009) and lower than in the non-Finnish European population (0.016) according to gnomAD. However, we have excluded possible genotyping errors by double checking the raw data and replicating 40 randomly chosen samples. Similarly to the Guilherme et al. report, we did not observe any rs1805065 T/T genotype carrier in either the athlete or control group, while T/C individuals were found among both controls and all athlete subgroups (endurance, power, and combat). Our study did not confirm the previous Brazilian observation that the minor allele T is negatively associated with the development of sport-related phenotypes [2]. Interestingly, our analyses reveal a positive association of minor allele T with success in combat sports.

Of note, similarly to the previous report, we also did not observe T allele in the high elite power group, which indeed agrees with the observation of T allele negatively associated with power phenotypes and its implication broadly discussed by Guilherme et al. 2019 [2]. Only two participants from the power group (*n* = 265) were carriers of T allele compared to 16 from the control group (*n* = 992). In that context, it is interesting to note the statistically significant higher frequency of allele T in high elite combat group compared to the control group (*p* = 0.018). Moreover, male high elite athletes (all sport groups combined) showed a higher T allele frequency than male controls (*p* = 0.036) but this effect was most likely due the high frequency in the combat sport subgroup (four carriers, while none among power athletes and two among endurance ones). Thus the rare allele T of rs1805065 (*SLC6A2*) seems to favor athletes in martial arts when in power competence can have an opposite effect.

An association of the intron variant rs2635438 of *SYNE1* with endurance athlete status at the GWAS level of significance (1.91 × 10^−8^) has been reported in a group of international-level athletes and controls from the general European population and validated in a group of elite Russian athletes and controls [10,11]. The results obtained from 753 European international-level athletes representing different sports disciplines were then validated in 219 elite athletes and 173 controls from the Russian population. The rs2635438 G allele was under-represented in 56 elite Russian long-distance athletes compared to 43 elite Russian sprinters (3.6 vs. 8.1%; OR = 0.132; SE = 0.9004; *p* = 0.024). Additionally, authors determined that enriched metabolic pathways associated with the rs2635438 result in a significant change in γ-glutamyl amino acid and glutamate metabolic pathways but a direct link of metabolite with SNPs remains unclear.

It is of note that the *SYNE1* intronic variants putatively affecting the clinical phenotype of patients with myofibrillar myopathy were recently identified by us [17]. It can be hypothesized that even variants with well-established involvement in the biology of the brain could be a “Janus-faced” variants with additional modulatory influence on the musculoskeletal system as well, with both functions putatively influencing athletic performance.

In our study, the minor allele frequency of rs2635438 was slightly higher in the athlete group compared to the control group (MAF = 0.041 vs. 0.032) but this difference did not reach statistical significance in any of the models tested and any pair-wise comparison.

## 5. Conclusions

In conclusion, we confirmed the association of the *SLC6A2* rs1805065 variant with athletic performance. The association of *SYNE1* rs2635438 did not reach statistical significance. Both results provide a valuable insight into the genetic architecture of psychological traits related to sport and allow several hypotheses to be put forward for further confirmation. It should be noted that even non-replicated single SNP association studies may still provide important clues to molecular mechanisms involved in the phenotypic expression of genetic background [18].

## Figures and Tables

**Table 1:** Characteristics of study groups.

	Samples No.	High Elite *	Elite **	Subelite ***	Male	Female
All sports	890	242	291	357	646	244
*Combat*	305	73	64	168	253	52
*Endurance*	320	104	126	90	214	106
*Power*	265	65	101	99	178	86
Control subject	1009	-	-	-	545	464

* High elite: Olympic/World Championships medalists and participants; ** Elite: multiple National Championships medalists and representatives at continental European level; *** Subelite: members of national teams, medalists of National Championships.

**Table 2:** Allele and genotype distribution.

Groups	Genotypes rs1805065	No. of rs1805065 Genotype	Comparisons vs. Controls *	OR	95%CI		*p* †	Genotypes rs2635438	No. of rs2635438 Genotype	Comparisons vs. Controls *	OR	95%CI		*p* †
All sports	CC	875	TT + CT vs. CC	1.0629	0.5224	2.1624	0.8664	TT	829	CC + TC vs. TT	0.8016	0.5649	1.1374	0.2146
	CT	15	TT vs. CT + CC	na	na	na	na	TC	58	CC vs. TC + TT	0	na	na	0.1846
	MAF	0.0084	T vs. C	1.0623	0.5237	2.1549	0.8669	MAF	0.0326	C vs. T	0.7880	0.5599	1.1089	0.1707
Power	CC	263	TT + CT vs. CC	0.4715	0.1077	2.0634	0.3070	TT	244	CC + TC vs. TT	0.9860	0.5979	1.6261	0.9561
	CT	2	TT vs. CT + CC	na	na	na	na	TC	21	CC vs. TC + TT	0	na	na	0.4683
	MAF	0.0037	T vs. C	0.4735	0.1085	2.0657	0.3087	MAF	0.0396	C vs. T	0.9618	0.5901	1.5679	0.8760
Endurance	CC	315	TT + CT vs. CC	0.9841	0.3577	2.7078	0.9753	TT	299	CC + TC vs. TT	0.6897	0.4071	1.1684	0.1650
	CT	5	TT vs. CT + CC	na	na	na	na	TC	18	CC vs. TC + TT	0	na	na	0.4276
	MAF	0.0078	T vs. C	0.9843	0.3591	2.6974	0.9754	MAF	0.0283	C vs. T	0.6812	0.4060	1.1431	0.1438
Combat	CC	297	TT + CT vs. CC	1.6700	0.7077	3.9408	0.2368	TT	286	CC + TC vs. TT	0.7611	0.4539	1.2762	0.2993
	CT	8	TT vs. CT + CC	na	na	na	na	TC	19	CC vs. TC + TT	0	na	na	0.4365
	MAF	0.0131	T vs. C	1.6611	0.7075	3.9003	0.2390	MAF	0.0311	C vs. T	0.7495	0.4515	1.2443	0.2633
Controls	CC	992						TT	928					
	CT	16						TC	79					
	TT	0						CC	2					
	MAF	0.0079						MAF	0.0411					
All sports high elite	CC	235	TT + CT vs. CC	1.8468	0.7512	4.5401	0.1749	TT	224	CC + TC vs. TT	0.8183	0.4695	1.4265	0.4789
	CT	7	TT vs. CT + CC	na	na	na	na	TC	16	CC vs. TC + TT	0	na	na	0.4900
	MAF	0.0145	T vs. C	1.8344	0.7505	4.4839	0.1769	MAF	0.0333	C vs. T	0.8039	0.4663	1.3859	0.4313
Combat high elite	CC	69	TT + CT vs. CC	3.5942	1.1698	11.0433	0.0406 ‡	TT	68	CC + TC vs. TT	0.8424	0.3303	2.1482	0.7193
	CT	4	TT vs. CT + CC	na	na	na	na	TC	5	CC vs. TC + TT	0	na	na	0.7034
	MAF	0.0274	T vs. C	3.5211	1.1618	10.6715	0.0414 ‡	MAF	0.0342	C vs. T	0.8267	0.3299	2.0715	0.6843
All sport male high elite	CC	168	TT + CT vs. CC	3.2024	1.0193	10.0610	0.0459 ‡	TT	163	CC + TC vs. TT	0.8423	0.4098	1.7313	0.6403
	CT	6	TT vs. CT + CC	na	na	na	na	TC	10	CC vs. TC + TT	0	na	na	0.5729
	MAF	0.0172	T vs. C	3.1637	1.0138	9.8735	0.0466 ‡	MAF	0.0289	C vs. T	0.8239	0.4062	1.6714	0.5910
Combat male high elite	CC	53	TT + CT vs. CC	6.7673	1.8513	24.7379	0.0099 ‡	TT	53	CC + TC vs. TT	1.0362	0.3555	3.0200	0.9480
	CT	4	TT vs. CT + CC	na	na	na	na	TC	4	CC vs. TC + TT	0	na	na	0.7462
	MAF	0.0351	T vs. C	6.5576	1.8228	23.5915	0.0103 ‡	MAF	0.0351	C vs. T	1.0067	0.3527	2.8734	0.9900

* All but male high elite comparisons were against control group. All sport male high elite and combat male high elite were analyzed against male control group; † Chi-square test; ‡ Fisher’s exact test.

**Table 3:** Multivariate logistic regression analysis of independent factors associated with high elite martial athlete performance as dependent variable (high elite martial athletes vs. controls).

Independent Variable	OR	95% CI		*p*
Sex (male vs. female)	3.090146	1.745723	5.469941	0.000106
rs1805065 (CT vs. CC)	3.961866	1.253152	12.52552	0.018939

## Data Availability

Data sharing not applicable.
